# Post-translational knockdown and post-secretional modification of EsxA determine contribution of EsxA membrane permeabilizing activity for mycobacterial intracellular survival

**DOI:** 10.1080/21505594.2020.1867438

**Published:** 2021-01-11

**Authors:** Yanqing Bao, Lin Wang, Jianjun Sun

**Affiliations:** Department of Biological Sciences and Border Biomedical Research Center, University of Texas at El Paso, El Paso, Texas, USA

**Keywords:** Mycobacterium tuberculosis, mycobacterium marinum, esxa, esxb, membrane-permeabilizing activity, intracellular survival

## Abstract

Current genetic studies (e.g. gene knockout) have suggested that EsxA and EsxB function as secreted virulence factors that are essential for *Mycobaterium tuberculosis* (Mtb) intracellular survival, specifically in mediating phagosome rupture and translocation of Mtb to the cytosol of host cells, which further facilitates Mtb intracellular replicating and cell-to-cell spreading. The EsxA-mediated intracellular survival is presumably achieved by its pH-dependent membrane-permeabilizing activity (MPA). However, the data from other studies have generated a discrepancy regarding the role of EsxA MPA in mycobacterial intracellular survival, which has raised a concern that genetic manipulations, such as deletion of *esxB-esxA* operon or RD-1 locus, may affect other codependently secreted factors that could be also directly involved cytosolic translocation, or stimulate extended disturbance on other genes’ expression. To avoid the drawbacks of gene knockout, we first engineered a *Mycobacterium marinum* (Mm) strain, in which a DAS4+ tag was fused to the C-terminus of EsxB to allow inducible knockdown of EsxB (also EsxA) at the post-translational level. We also engineered an Mm strain by fusing a SpyTag (ST) to the C-terminus of EsxA, which allowed inhibition of EsxA-ST MPA at the post-secretional level through a covalent linkage to SpyCatcher-GFP. Both post-translational knockdown and functional inhibition of EsxA resulted in attenuation of Mm intracellular survival in lung epithelial cells or macrophages, which unambiguously confirms the direct role of EsxA MPA in mycobacterial intracellular survival.

## Introduction

Pathogenic mycobacteria, like *Mycobacterium tuberculosis* and *Mycobacterium leprae* species, have been imposing great threats to public health for decades [1,2]. Extensive research on their virulence and related phenotypes is urgently needed to minimize the impacts of pathogenic mycobacteria. Completion of whole genome sequencing of multiple mycobacteria species has facilitated researches on mycobacterial pathogenicity, epidemiology, detection, and vaccine development [3–9]. Various gene editing methods allow researchers to explore genes of interest for their roles in mycobacterial virulence [10].

The *esxB-esxA* operon is located within the *esx-1* locus in Mtb genome that encodes a Type VII secretion system [11]. Current genetic studies (e.g. gene deletion, disruption, or mutation) have suggested that EsxA and EsxB play an essential role in mycobacterial virulence, pathogenicity, intracellular survival, and escape from immune responses [12–17]. EsxA and EsxB are secreted as a heterodimer through the ESX-1 secretion system [18]. Our previous studies have demonstrated that EsxA, but not EsxB, exhibits acidic pH-dependent MPA [19]. The MPA is uniquely present in the EsxA from pathogenic Mtb and Mm, but is absent in the highly homologous EsxA from nonpathogenic *Mycobacterium smegmatis* (Ms) [19,20]. Considering that EsxA of Mm and Mtb share over 90% sequence identity, and Mm’s virulence could be restored with MtbEsxA [21], it supports that they are functionally exchangeable, and Mm is a surrogate model for research on EsxA. Moreover, this also suggests that EsxA MPA is one of the key factors determining the virulence phenotype of mycobacteria. This notion is further confirmed by our recent study showing that single-residue mutations Q5V and Q5K in EsxA either up or down-regulated the MPA and consequently up or down-regulated the cytosolic translocation, intracellular survival, and virulence of Mtb and Mm in cultured macrophages and in zebrafish [21]. Most recently, we have found that the N^α^-acetylation of EsxA at the residue T2 is required for EsxAB heterodimer separation, a prerequisite for EsxA to permeabilize membranes. The non-acetylated mutations at T2 (e.g. T2A and T2R) inhibit the acidic pH-dependent heterodimer separation and consequently attenuate Mm cytosolic translocation and survival in macrophages [22]. Therefore, current studies have well established that EsxA’s MPA contributes to mycobacterial intracellular survival by mediating cytosolic translocation.

However, EsxA’s necessity has been challenged by the codependency among effectors of ESX-1. Studies conducted with classical gene-knockout strains indicate that, without functional EsxA/B, the expression or secretion of other ESX-1 effectors (e.g. EspA, EspC, or EspB) would be affected to various extends [18,23–26]. Although the mechanism of how these effectors contribute to mycobacterial intracellular survival or virulence has not been elucidated yet, the application of *esxB-esxA* knockout strain potentially blurs the exact roles of either EsxA/B or other effectors. As shown in a recent study, a collection of Mm ESX-1 transposon mutants that disrupt EsxA secretion could still infect macrophages and permeabilize phagosomes, suggesting that other factors or mechanisms independent of EsxAB play a role in cytosolic translocation [17]. Plus, it has been reported that loss of specific genes or locus, even if they are not regulators, leads to global disturbance on bacterial transcriptome [27–29], which potentially produces artificial changes of phenotypes. Combined together, it has aroused concern about classical gene deletion methods in research of specific ESX-1 genes.

In order to overcome the drawback of gene knockout and to determine the exact role of EsxA in mycobacterial intracellular survival, in the present study we employed two approaches to avoid the potential side effects caused by gene knockout. We first constructed an Mm recombinant strain, namely Mm(EsxB-DAS4+), in which a degradation signal peptide DAS4+ was fused to the C-terminus of EsxB for conditional knockdown of EsxB [30,31]. Secondly, we engineered another Mm strain, namely Mm(EsxA-ST) by fusing an ST to the C-terminus of EsxA. The EsxA-ST can be specifically recognized by SpyCatcher (SC) to inhibit its MPA. The results obtained in the present study have shown that both post-translational knockdown and functional inhibition of EsxA attenuated Mm intracellular survival.

## Materials and methods

### Bacterial strains, cell lines, plasmids and primers

The *Mycobacteria marinum* M strain [32], type I human lung epithelial cell line WI-26 VA4 and human monocyte cell line THP-1 were purchased from American Type Culture Collection (ATCC, Manassas, VA, USA) and preserved in our lab [33,34]. Type II human lung epithelial cell line A549 was kindly provided by Dr. Jianying Zhang at The University of Texas at El Paso [35].

The pJSC407, pGOAL17, and pGMCKq1-10M1-sspBopt plasmids were kindly provided by Dr. Hugues Ouellet at The University of Texas at El Paso. The pJSC407 and pGOAL17 plasmids were used to produce the pJSC407-sacB suicide plasmid. The pGMCKq1-10M1-sspBopt was used for inducible expression of adaptor protein SspB for DAS4+ inducible knockout system. The pQE80L-SpyCatcher-ELP-GFP plasmid was purchased from Addgene (#69,835, Watertown, MA, USA) for prokaryotic expression and purification of SpyCatcher-ELP-GFP protein.

Since the ELP linker between SC and GFP was found cytotoxic to human cells [36], we removed it and inserted SpyCather (SC) into pcDNA3-EGFP vector for eukaryotic expression of SpyCatcher-GFP (SC-GFP) in A549. However, we found that transient transfection of pcDNA3-SC-GFP had a very low expression of SC-GFP in several mycobacteria susceptible cell lines, including WI-26, RAW264.7, THP-1, and A549. In order to enhance SC-GFP expression in eukaryotic cells, the coding sequence of SC was optimized for expression in human cell lines and synthesized by ThermoFisher Scientific (Rockford, IL, USA). The codon-optimized SC sequence was inserted into pcDNA3-EGFP (#13,031, Watertown, MA, USA) for eukaryotic expression of SC-GFP. Even after optimization, SC-GFP was efficiently expressed only in A549 cell line.

The primers for site-directed mutagenesis, identification, and RT-qPCR were all designed according to Mm’s genomic DNA sequence (GenBank Accession Number: CP000854.1) and synthesized by Sigma-Aldrich (St. Louis, MO, USA). The primers used in this study are listed in Table 1.

**Table 1.** Plasmids and primers used in this study

Primers	Sequences
EsxB-D4-UF	TGCTCTAGAGACCAACTTCTTCGGCATCAA
EsxB-D4-UR	CTACGACGCGTCGGCGTAGTTTTCGCTGTAATTCTCATCGTTCGCGGCGAAGCCCATTTGCGAGGAC
EsxB-D4-DF	GCCGCGAACGATGAGAATTACAGCGAAAACTACGCCGACGCGTCGTAGTTCCCCTAAAACGATAAAGAAACG
EsxB-D4-DR	TGCTCTAGAGGTTGCGTGGGCCTGTTC
EsxB-D4-OP-F	TACAGCGAAAACTACGCCGAC
EsxB-D4-OP-R	ATTCTACGCGAACGAGAGGG
EsxA-ST-UF	TGCTCTAGAAAGACGACTGGGATGACGAG
EsxA-ST-UR	CTACTTAGTTGGCTTGTAAGCATCAACCATGACAATATGAGCGCCCGAGCGAGCAAACATCCCCGTGAC
EsxA-ST-DF	CGCTCGGGCGCTCATATTGTCATGGTTGATGCTTACAAGCCAACTAAGTAGTCCCCCCTCTCGTTCGC
EsxA-ST-DR	TGCTCTAGAGCCGGTTCTTCTGCTATGTC
EsxA-ST -OP-F	GCTCATATTGTCATGGTTGATGC
EsxA-ST -OP-R	ACGCGGTACCAGGATCAAAG
EsxB-D4 qRT F	CAACAAGCAGAAGGCCGAAC
EsxB-D4 qRT R	TCGGCGTAGTTTTCGCTGTA

To fuse ST and DAS4+ to the respective C-terminus of EsxA and EsxB, the homologous arms that flank the genes *esxA* or *esxB* were amplified from the genomic DNA of Mm, respectively. Then the amplified fragments were inserted into pJSC407-sacB. Similarly as previously described [21], the recombinant suicide plasmid was electroporated into competent Mm(WT). After selection with 2% sucrose on the 7H10 plates (10% OADC), the colonies were picked to culture until OD600 reached 0.6 to 0.8. Then, the culture pellet was collected, resuspended in ultrapure water, and heated at 95°C for 5 min. The heated pellet was used as PCR template and identified with primers that span the target genes (*esxA or esxB*) and the tags. The positive colonies were named as Mm(EsxB-DAS4+) and Mm(EsxA-ST), respectively. The plasmid pGMCKq1-10M1-sspBopt was electroporated into Mm(EsxB-DAS4+) to obtain Mm(EsxB-DAS4+)|pGMCKq1 for inducible knockdown of EsxB protein by ATC. All the Mm strains used in this study carry a mCherry-coding plasmid.

### Western blot and immunofluorescence assay

To detect the expression and secretion of EsxB-DAS4+ or EsxA-ST, the culture lysate (CL) and culture filtration were (CF) prepared similarly as reported elsewhere [23]. Briefly, the strains were first cultured in 7H9 media (10% OADC, 0.05% Tween-80) until OD_600_ reached 0.8 ~ 1.0. Then, the culture pellet was collected by centrifugation and washed twice with Sauton media. After that, the culture was diluted in Sauton media (0.05% Tween-80) at OD_600_ = 0.1 and cultured at 30°C, 150 rpm for 72 h. After centrifugation at 15,000 rpm at 4°C for 1 h, the bacterial pellet and culture supernatant were collected separately. For the pellet, it was first resuspended with certain volume of gel filtration buffer (150 mM NaCl, 20 mM Tris, pH 7.4) and lysed in a micro-beads beater. After centrifugation, the supernatant was collected and mixed with four volumes of ice-cold acetone overnight at −20°C. The precipitated protein was collected as CL samples. For the cultural supernatant, it was concentrated with ultra-filtration tubes (Sartorius, Bohemia, NY, USA) by 200-fold at 4°C. The concentrated samples were used as CF. Protease inhibitor cocktail (Pierce, ThermoScientific, Rockford, IL, USA) was added to all samples.

To detect the inducible knockdown of EsxB, Mm(EsxB-DAS4+)|pGMCKq1 was first cultured in 7H9 media (10% OADC, 0.05% Tween-80) until OD_600_ reached 0.8 ~ 1.0. Then, the culture was diluted again in 7H9 (10% OADC, 0.05% Tween-80) with 500 ng/ml of ATC at OD_600_ = 0.02. The culture pellet was then collected at various times of post-induction to produce CL as mentioned above. In all Western blots, the expression or secretion of EsxB, EsxA, EsxB-DAS4+, and EsxA-ST were detected using anti-EsxB polyserum (NR-19,361, BEI Resources, Manassas, VA, USA) and anti-EsxA antibody (HYB076-08, Santa Cruz Biotechnology, Dallas, TX, USA), respectively. The GroEL and Ag85B were detected using anti-GroEL antibody (NR-13,813, BEI Resources, Manassas, VA, USA) and anti-Ag85B complex polyserum (NR13800, BEI Resources, Manassas, VA, USA), respectively. All samples were loaded according to the same pellet weight.

Immunofluorescence assay was also used to test the inducible knockdown of EsxB-DAS4 +. As described above, the Mm(EsxB-DAS4+)|pGMCKq1 pellet was collected at 48 h of post ATC induction. After the bacteria were fixed to the coverslip, the bacteria were incubated with 2% BSA for 30 min at RT. After that, anti-EsxB polyserum and anti-EsxA polyserum (NR-13,803, BEI Resources, Manassas, VA, USA) were used as a primary antibody, and the FITC-labeled secondary antibody was used to visualize bacteria-associated EsxB-DAS4+ and EsxA under confocal microscopy (LSM 700, Zeiss, San Diego, CA, USA). To quantify the amount of bacteria-associated EsxB-DAS4+ and EsxA, the layers of FITC (green) and mCherry (red) were extracted from each image to calculate the overlap rate between the green and red signal. Briefly, the areas with green or red signals were first calculated. Then, the area of green that was colocalized with red areas was calculated. The area’s percentage relative to the total red area is the overlap rate we need, which was calculated according to equation in Table 2. Layer extraction and area calculation were achieved with Python 3.7.3 [37].

**Table 2.** Equations used for fluorescence image analysis and hemolysis assay

Knockdown of EsxB-DAS4 + ^a^	$\frac{areaG\cap^{a}reaR}{areaG}$
SC-GFP’s effect on Mm(EsxA-ST) ^a^	$\frac{areaR\cap^{a}reaG}{areaG}$
Hemolysis (%)	$\frac{A405Sample-A405NegativeControl}{A405PositiveControl-A405NegativeControl}$

^a^: areaG, area of green fluorescence signal; areaR, area of red fluorescence signal.

To detect the covalent binding between SC-GFP and EsxA-ST, the total lysate of Mm(EsxA-ST) was incubated with purified SC-GFP at RT for 45 min. The mixture was applied to SDS-PAGE and transferred to PVDF membrane, which was followed by Western blots using anti-EsxA antibody and anti-GFP McAb (4B10, Cell Signaling Technology, Danvers, MA, USA), respectively. The purified EsxA was incubated with SC-GFP as a control.

To verify if SC-GFP could bind to the bacteria-associated EsxA-ST specifically, the culture pellet of Mm(EsxA-ST) was collected and incubated with the purified SC-GFP at RT for 45 min. After several washes with PBS, the pellet was added into a 24-well plate with round coverslips. After a brief centrifugation, the bacteria were attached to the coverslips and then fixed with 4% paraformaldehyde. The fixed bacteria were observed with confocal microscopy. The Mm(WT) was used as a control.

To test the effects of the endogenously expressed SC-GFP on Mm(EsxA-ST) intracellular survival, A549 cells were transiently transfected with pcDNA3-SC-EGFP or pcDNA3-EGFP at 800 ng/well, respectively. At 24 h post-transfection, the cells were infected with Mm(WT) or Mm(EsxA-ST) at MOI = 2. At 24 hpi and 48 hpi, the cells in the randomly selected fields were imaged and the intracellular survival was calculated as the ratio of mCherry (red): EGFP (green). Same as above, the areas with green or red signals were first calculated. Then, the red areas that were colocalized with green were calculated. This area percentage in the total green area is the overlap rate we need, which was calculated according to equation in Table 2. Layer extraction and area calculation were achieved with Python 3.7.3 [37].

### Liposome leakage assay

As previously described [21], the liposome was produced from 1, 2-Dioleoyl-sn-glycero-3-phosphocholine (DOPC) lipid film. To encapsulate dye/quencher pairs, 8-aminonapthalene-1,3,6 trisulfonic acid (ANTS)/p-xylene-bis-pyridinium bromide (DPX) and DOPC film were rehydrated in 50 mM HEPES (pH 7.3) for six freeze-thaw cycles. Then, the mixture was filtered through 200 nM polycarbonate membrane (Avanti Polar Lipids, Alabaster, AL, USA) for 20 times. After that, the redundant salt in mixture was removed in a G-25 desalting column (GE Healthcare Life Sciences, Pittsburgh, PA, USA) equilibrated with 50 mM HEPES solution (pH 7.3).

The dequenching of ANTS fluorescence was measured in an ISS K2 multiphase frequency and modulation fluorometer (ISS, Champaign, IL, USA) with excitation at 380 nm and emission at 520 nm. 100 μl of liposome and 100 μg of protein were mixed with the assay buffer (150 mM NaCl, 20 mM Tris, pH7.4) to a final volume of 1350 μl. After 30 sec of incubation, 150 μl of 1 M NaAc solution (pH 4.0) was added into the mixture to activate acidic pH-dependent membrane insertion, and the fluorescence intensity was recorded continuously for the following 180 sec. The mixture was continuously stirred throughout the assay. Prior to the assay, EsxA-ST was incubated with SC-GFP (molar ratio 1:0.5) for 2 hours at RT to allow formation of the covalent bond between EsxA-ST and SC-GFP.

### Hemolysis assay

To verify if SC-GFP binding could decrease hemolysis ability of Mm(EsxA-ST), we used sheep red blood cells (RBC, MP, Solon, OH, USA) as the model. As reported elsewhere [38], the Mm(EsxA-ST) was cultured in 7H9 media (10% OADC, without Tween-80) until OD_600_ reached at least 1.0. The culture pellet was collected to wash twice with PBS, and the dry culture pellet was weighted. After that, the pellet was resuspended in 5 ml of PBS and divided into two equal parts. SC-GFP was added into one part at 1 μg/mg of culture pellet and incubated at RT for 45 min. Another part was incubated with PBS as a non-treated control. Then, the pellet was washed 3 times with PBS to remove residual SC-GFP. The pellet was weighted again and resuspended with certain volume of PBS to reach OD_600_ around 1.0. After that, 3 ml of the resuspended pellet was concentrated into 100 μl of PBS. 100 μl of 1% RBC was gently mixed with 100 μl of the resuspended mycobacteria above in 1.5 ml centrifuge tubes and centrifuged at 3200 × g for 5 min. After incubation at 33°C for 2 hr, the samples were resuspended and centrifuged at 3200 × g for 5 min again. 100 μl of the supernatant was collected for absorbance measurement at 405 nm.

Mm(WT) and Mm(ΔEsxA:B) were included in the assay, each strain was treated as above. PBS was used as a blank control, and 0.1% Triton X-100 PBS solution was used as a positive control. Hemolysis percentage of each strain was calculated according to equation in Table 2.

### RNA extraction and RT-qPCR

The Mm(EsxB-DAS4)|pGMCK1q cells were treated with or without ATC and were collected from 5 ml liquid culture at 24 and 48 h post-induction. Total RNA was extracted with TRI reagent (Ambion, Carlsbad, CA, USA). Total RNA was subjected to reverse transcription with High Capacity cDNA Reverse Transcription Kit (ThermoFisher Scientific, Rockford, IL, USA) at 25°C for 10 min, 37°C for 120 min, then 85°C for 5 min for cDNA templates. SYBR® Select Master Mix kit (ThermoFisher Scientific, Rockford, IL, USA) was used for RT-PCR, according to the manufacturer’s instruction: 1 μl cDNA, 1 μl forward or backward primer (10 μM), 7 μl nuclease-free water, and 10 μl SYBR master mix were added. Reactions were on the Step One Real-Time PCR System (Applied Biosystems, USA) at 95°C for 20 s, 40 cycles at 95°C for 3 s, 60°C for 30 s, and a melting curve. The target genes were tested in triplicate and the rpoB gene was used as the internal control. Primers (EsxB-DAS4+ qRT F/R, Table 1) were designed to span the regions of the coding sequences of EsxB and the DAS4+ tag, based on National Center for Biotechnology Information (NCBI) Primer-BLAST [39]. Relative transcription levels of *esxB-DAS4+* in ATC treated group according to that of non-ATC treated group were calculated with the 2^−∆∆Ct^ method [40].

### Lactate dehydrogenase (LDH) release assay

To evaluate ATC’s cytotoxicity effect on cells, WI-26 was cultured with different concentrations of ATC addition. After 48 h, the media was collected for LDH release assay (CytoTox96 Non-Radioactive Cytotoxicity Assay, Promega, Maison, WI, USA) according to the manufacturer’s protocol. The cells without ATC addition were used as a blank control, while the cells lysed with 0.1% Triton X-100 were used as maxim release. LDH release percentage was calculated according to the equation in the protocol.

### Mycobacterial intracellular survival

To test Mm(EsxB-DAS4+)’s intracellular survival in WI-26 cells, the cells were plated in 24-well plates at 2 × 10^5^ cells per well and cultured for 24 h. The single cell preparation of Mm(WT), Mm(EsxB-DAS4+), and Mm(ΔEsxA:B) were added into each well at MOI = 2, respectively. After a brief centrifugation, the plates were incubated at 30°C, 5% CO_2_ for 45 min. After that, cells were washed with PBS to remove free bacteria. Eagle’s minimum essential media (EMEM) with 100 μg/mL Amikacin and 2% fetal bovine serum (FBS) was added to kill extracellular mycobacteria for 2 h. Then, the media was replaced with EMEM containing 50 μg/ml Amikacin, 2% FBS and cultured at 30°C, 5% CO_2_. At 2, 24, and 48 hpi, the cells were lysed with 0.1% Triton X-100 and spread on 7H10 plates (10% OADC) for colony-forming unit (CFU) counting. The CFU data at 24 hpi and 48 hpi were calculated into the percentage based on the data at 2 hpi.

To test the conditional knockdown strains’ intracellular survival in WI-26, first the culture pellet of Mm(EsxB-DAS4+)|pGMCKq1 and Mm(WT) was induced with ATC as described above. The pellet of both induced and uninduced groups was collected to produce single cell preparation. Then, the WI-26 cells were cultured and infected as described above, and the CFU at 48 hpi was quantified. To test the effect of intracellular knockdown, the WI-26 cells were first infected with Mm(EsxB-DAS4+)|pGMCKq1 and Mm(WT), respectively. After killing the extracellular mycobacteria (2 hpi), the media was replaced with EMEM containing 5 μg/ml ATC, 50 μg/ml Amikacin, 2% FBS and cultured at 30°C, 5% CO_2_. It has been reported that most of intracellular mycobacteria escape from phagosome at around 20 hpi [41,42], so the CFU at 24 hpi and 48 hpi were collected for analysis.

Since it has been reported that Mm(WT) ruptures phagosome and translocates into the cytosol between 24 and 48 hpi in THP-1 cells, we also used THP-1 to test the effect of intracellular knockdown. The THP-1 cells were planted in 24-well plates at 1 × 10^6^ and stimulated with 100 nM of 12-O-Tetradecanoylphorbol-13-acetate (PMA) overnight [43]. After that, the differentiated THP-1 cells were rested for 24 h before they were infected with mycobacteria at MOI = 1 for 4 h. After killing the extracellular bacteria (2 hpi), ATC was added into the experimental groups (5 μg/mL) to induce EsxB-DAS4+ degradation. Mm(WT) was used as a control. The CFU of each group was collected at 24 hpi and 48 hpi for analysis.

Since A549 is the only cell line with a high transfection efficiency, it was used for Mm(EsxA-ST)’s intracellular survival assay. The cells were plated in 24-well plates at 1 × 10^5^ cells per well and cultured with F-12 K containing 10% FBS for 24 h. Then, the cells were infected with Mm(WT), Mm(EsxA-ST), and Mm(ΔEsxA:B) in the same way as in WI-26 cells. At 2, 24, and 48 hpi, the cells were lysed with 0.1% Triton X-100 and spread on 7H10 plates (10% OADC) for CFU counting. The CFU data at 24 hpi and 48 hpi were calculated as the percentage relative to the data at 2 hpi.

To test if SC-GFP could inhibit Mm(EsxA-ST)’s intracellular survival, the cells were transiently transfected with pcDNA3-SC-EGFP and pcDNA3-EGFP. The cells were then infected with Mm(WT) and Mm(EsxA-ST) at MOI = 2. At 24 hpi and 48 hpi, the cells were lysed with 0.1% Triton X-100 to collect the CFU data for analysis.

## Result

### Mm(EsxB-DAS4+) has a reduced expression and secretion of EsxB-DAS4+, but it still exhibits a higher intracellular survival than Mm(ΔEsxA:B)

**Figure 1.**
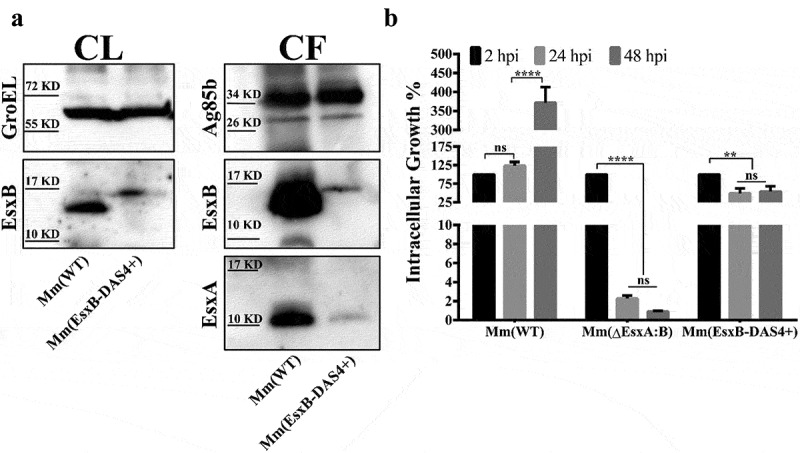
Mm(EsxB-DAS4+) has a reduced expression and secretion of EsxB-DAS4+, but it still exhibited a higher intracellular survival than Mm(ΔEsxA:B). (a) The culture filtrates (CF) and cell lysates (CL) of Mm(WT) and Mm(EsxB-DAS4+) were applied to SDS-PAGE, and the expression and secretion of EsxB or EsxB-DAS4+ were detected with Western blots by using anti-EsxB serum. The secretion of EsxA in CF was detected with anti-EsxA antibody. GroEL and Ag85B were also detected as the controls for CF and CL, respectively. (b) WI-26 cells were infected with Mm(WT), Mm(EsxB-DAS4+) and Mm(ΔEsxA:B) at MOI = 2, respectively. At 2, 24 and 48 hpi, the cells were lysed for CFU counting. For each strain, the CFU at 24 and 48 hpi was calculated to the percentage of the CFU at 2 hpi. The experiment was replicated three times and the data is presented as mean ± SD. The statistical analysis was performed with One-way ANOVA method, followed by Holm-Sidak multiple comparisons. **P< 0.01, ****P< 0.0001

To avoid the potential artifacts caused by gene knockout, we set off to construct an Mm strain that allows inducible knockdown of EsxA at the post-translational level. Initially, we attached the degradation peptide DAS4+ to the C-terminus of EsxA on the Mm chromosome. However, we failed to acquire this engineered strain due to unknown reasons. Since earlier studies have shown that intact C-terminal is critical for EsxB’s interaction with other ESX-1 proteins [44–46], while some C-terminal tags have no significant impact on EsxB [47], we attached DAS4+ to the C-terminus of EsxB (**Fig. S1A**) and confirmed the construction by PCR (**Fig. S1B**). Western blot analysis showed that EsxB-DAS4+ was expressed at a reduced level than wild type EsxB in Mm(WT) in the culture lysates (Figure 1a). In the culture filtrate, EsxB-DAS4+ was significantly lower than EsxB, suggesting that the secretion of EsxB-DAS4+ was also affected by DAS4+ tag. It is also not surprising that EsxA secretion was also affected, which is consistent with the known fact that EsxA and EsxB are secreted as a heterodimer [44,48] (Figure 1a). As expected, the intracellular survival of Mm(EsxB-DAS4+) was attenuated and dropped to 50% at 24 hpi and 48 hpi, while Mm(WT) reached more than 350% at 48 hpi (Figure 1b). However, compared with Mm(ΔEsxA:B), whose survival rate dropped 1%, Mm(EsxB-DAS4+) still maintains a higher level of intracellular survival in WI-26 (Figure 1b).

### Addition of ATC induces knockdown of EsxB-DAS4+ at the post-translational level

**Figure 2.**
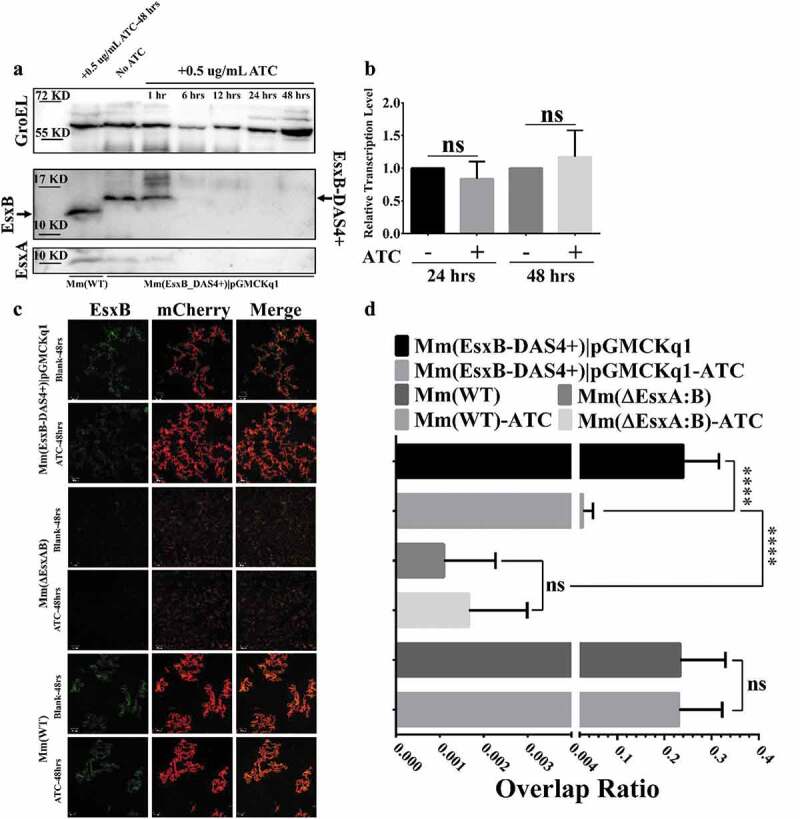
ATC-induced knockdown of EsxB-DAS4+ at the post-translational level. (a) The Mm(EsxB-DAS4+)|pGMCKq1 culture was treated without ATC or with ATC (0.5 μg/ml) for the indicated times. As a control, the Mm(WT) culture was also treated with ATC (0.5 μg/ml) for 48 h. The expression of EsxB-DAS4+ and EsxA were detected by Western blots. The bands of EsxB and EsxB-DAS4+ are designated with arrows. (b) The relative mRNA level of EsxB-DAS4+ after 24 h and 48 h of ATC treatment was determined by RT-qPCR. The data from the non-ATC treated group was used as a control. The experiment was replicated for three times and the data is presented as mean ± SD. The statistical analysis was performed with *t* tests. (c) The mCherry-expressing Mm(EsxB-DAS4+)|pGMCKq1 cells were treated without or with ATC (0.5 μg/ml) for 48 h. Then the bacteria were incubated with anti-EsxB serum, followed by FITC-labeled secondary antibody, to detect the bacteria-associated EsxB-DAS4 +. Images from all groups were taken under a LSM700 confocal fluorescence microscopy with the same configuration. For each strain, 12 random sights were taken from two replicate wells. The scale bar represents 50 µm. (d) The Green/Red overlap ratio in the randomly selected fields was quantified. The left fragment of X-axis ranges from 0 to 0.004, and the right fragment ranges from 0.02 to 0.4. The IFA assay were replicated for three times and the data is presented as mean ± SD. The statistical analysis was performed with One-way ANOVA method, followed by Holm-Sidak multiple comparison. ****< 0.0001

To test the inducible degradation of EsxB-DAS4+, the Mm(EsxB-DAS4+) liquid culture was treated with ATC (0.5 μg/ml) for various times. The culture lysate was applied to SDS-PAGE, followed by Western blot. The expression of EsxB-DAS4+ was significantly reduced after 6 h of ATC treatment, while ATC had no effect on EsxB even after 48 h treatment on Mm(WT) (Figure 2a). Interestingly, the expression of EsxA was also diminished after 6 h treatment (Figure 2a). Given that EsxA and EsxB reportedly form a heterodimer and share a codependent stability [25,49,50], it is reasonable to believe that the protease induces degradation on both of them. Moreover, ATC did not down-regulate the transcription level of *esxB-DAS4+* (Figure 2b), indicating the inducible degradation specifically targets at the post-translational level. Considering the limited detection sensitivity of the antibodies used in Western blot, we further determine the inducible degradation of EsxB-DAS4+ with an immunofluorescence assay. As expected, the ATC-treated Mm(EsxB-DAS4+)|pGMCKq1 cells showed significantly reduced EsxB-DAS4+ expression, compared to the non-ATC-treated cells, while ATC had no effect on EsxB expression in Mm(WT). As a negative control, Mm(ΔEsxA:B) didn’t show EsxB immunofluorescence signal and had a very low intracellular survival (Figure 2c**, D**). We also tested the effect of ATC on EsxA expression. As expected, the ATC had a similar effect on EsxA expression as EsxB (Figure 4s).

### ATC-induced knockdown of EsxB-DAS4+ attenuates Mm’s intracellular survival in mammalian cells

**Figure 3.**
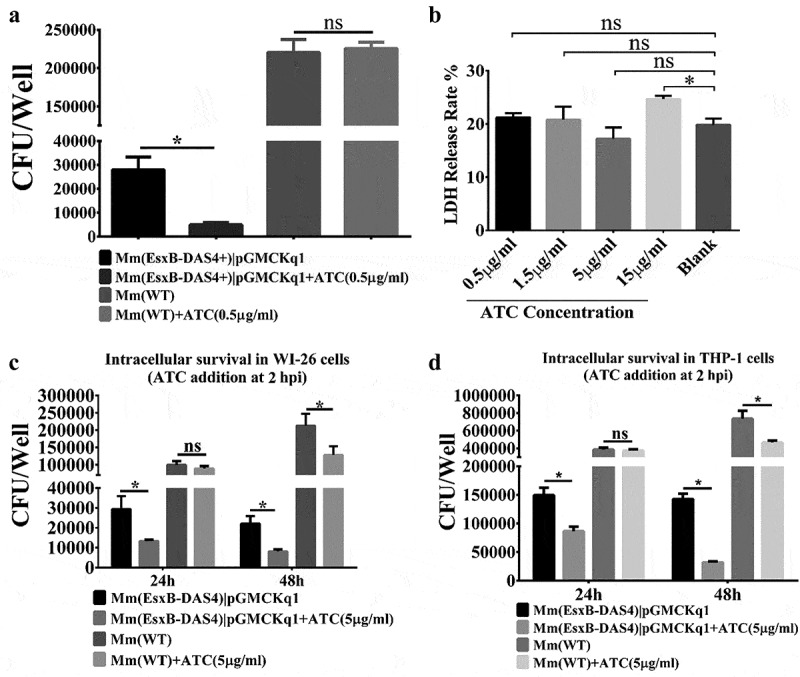
Inducible knockdown of EsxB reduced Mm intracellular survival. (a) Mm(EsxB-DAS4+)|pGMCKq1 and Mm(WT) were treated with/out ATC (0.5 μg/ml) for 48 h before single cell preparation. Then it was used to infect WI-26 cells at MOI = 2. At 48 hpi, the cells were collected and subjected to intracellular survival assay (CFU/well). The strains without ATC induction were used as controls. (b) WI-26 cells were treated with ATC at various concentrations for 48 h. The cytotoxicity was measured by LDH release assay. (c) WI-26 cells were infected with Mm(EsxB-DAS4+)|pGMCKq1 and Mm at MOI = 2. At 2 hpi, ATC (5 μg/ml) was added to the cell culture to induce degradation of EsxB-DAS4 +. At 24 and 48 hpi, the cells were harvested and subjected to intracellular survival assay (CFU/well). (d) Similarly, THP-1 cells were infected with Mm(EsxB-DAS4+)|pGMCKq1 or Mm at MOI = 1. At 2 hpi, ATC (5 μg/ml) was added to the cell culture to induce EsxB-DAS4+ degradation. At 24 and 48 hpi, the cells were harvested and subjected to intracellular survival assay (CFU/well). The cells without ATC addition were used as controls. The experiments were replicated for three times and the data is presented as mean ± SD. For cytotoxicity data, the statistical analysis was performed with One-way ANOVA, followed by Holm-Sidak multiple comparison. For CFU data, the statistical analysis was performed with multiple *t* test between ATC treated and nontreated groups of each strain. *< 0.05

We then tested the effects of ATC induction on Mm’s intracellular survival in WI-26 cells. The Mm(EsxA-DAS4+)|pGMCKq1 cells were pre-treated with ATC (0.5 μg/mL) and then produced into single cell preparation for infection of WI-26 cells. As expected, the ATC-treated Mm(EsxB-DAS4+)|pGMCKq1 had a significantly lower intracellular survival than that without ATC treatment. As control, the intracellular survival of Mm(WT) is not affected by ATC (Figure 3a). Since ATC could be added to WI-26 culture media, we set out to test the effects of ATC treatment on Mm intracellular survival during the infection. First, we titrated the cytotoxic effect of ATC and found that ATC did not have significant cytotoxicity at a concentration up to 5 μg/mL (Figure 3b). To ensure knockdown of EsxB when Mm(EsxB-DAS4+) is inside host cells, the highest concentration without cytotoxicity (5 μg/mL) was applied to cell culture medium after killing the extracellular bacteria (2 hpi). At 24 hpi and 48 hpi, the intracellular survival of Mm(EsxB-DAS4+)|pGMCKq1 with ATC addition was significantly lower than the group without ATC (Figure 3c). ATC had no effect on the intracellular survival of Mm(WT) at 24 hpi, but had a down-regulatory effect at 48 hpi. It could be because that ATC is an antibiotic that inhibits bacterial growth overtime, or it still has certain cytotoxic effects on the host cells. However, we noticed that the ATC unspecific inhibition to Mm(WT) was much less than its specific inhibition for Mm(EsxB-DAS4+) (Figure 3c). Since Mm has been shown to rupture phagosome with EsxA in THP-1 cells [42], we also tested ATC’s effect in THP-1. As shown in Figure 3d, similar results were acquired from THP-1 cell line. Together, knockdown of EsxB-DAS4+ by ATC either before or during the infection attenuates mycobacterial intracellular survival.

### Insertion of ST to the C-terminus of EsxA allows SC-GFP to covalently modify EsxA-ST at the post-translational level

**Figure 4.**
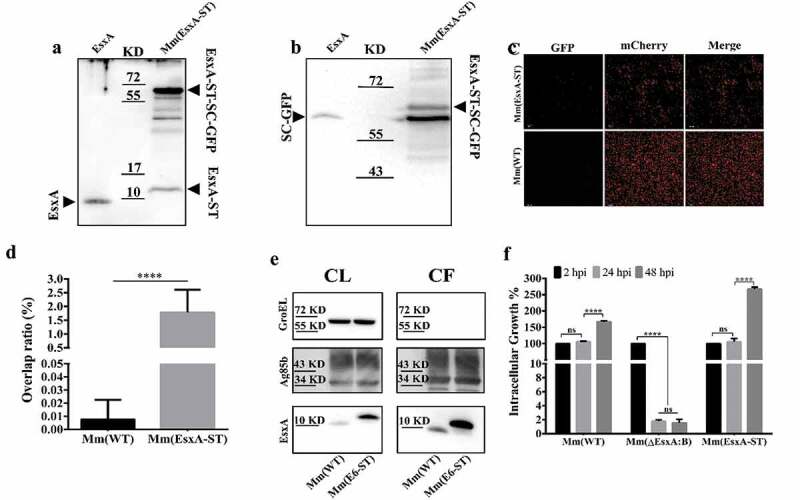
Insertion of ST at the C-terminus of EsxA didn’t affect Mm intracellular survival and allowed post-secretional labeling of EsxA by SpyCatcher(SC)-GFP. (a) The total lysate of Mm(EsxA-ST) were incubated with the purified SpyCatcher(SC)-GFP. The purified EsxA was incubated with SC-GFP as a control. The samples were subjected to Western blots. EsxA, EsxA-ST and EsxA-ST-SC-GFP were detected with anti-EsxA antibody. (b) SC-GFP and EsxA-ST-SC-GFP were detected with anti-GFP antibody. (c) The mCherry-expressing Mm(EsxA-ST) and Mm(WT) were incubated with SC-GFP. The images were taken under a LSM700 confocal fluorescence microscopy with the same configuration. For each strain, 12 random sights were taken from two replicate wells. The scale bar represents 20 µm. (d) The Green/Red overlap ratio in the randomly selected fields was quantified. The SC-GFP labeling assay was replicated for three times and the data is presented as mean ± SD. The statistical analysis was performed with *t*-test. ****< 0.0001. (e) The culture filtrates (CF) and cell lysates (CL) of Mm(WT) and Mm(EsxA-ST) were applied to Western blots, and the expression and secretion of EsxA or EsxA-ST were detected with anti-EsxA antibody. GroEL and Ag85B were detected as loading controls for CF and CL, respectively. GroEL was also detected in CF to make sure the EsxA-ST could be successfully secreted. (f) A549 cells were infected with Mm(WT), Mm(EsxA-ST) and Mm(ΔEsxA:B) at MOI = 2, respectively. At 2, 24 and 48 hpi, the cells were collected and subjected to intracellular survival tests. For each strain, the CFU of 24 and 48 hpi was counted and calculated to the percentage of the CFU at 2 hpi. The experiment was replicated for three times and the data is presented as mean ± SD. The statistical analysis was performed with One-way ANOVA method, followed by Holm-Sidak multiple comparisons. ****< 0.0001

When ST and SC are incubated together, a covalent amide linkage is automatically formed between a lysine on ST and an asparagine on SC [51,52]. The amide bond formation is fast, irreversible, and highly tolerant to various conditions [53], which makes it an ideal tool for protein modification. According to an early report, modification of EsxA N-terminus impaired its secretion and function [48], so we engineered the ST to C-terminus of EsxA on the Mm chromosome (**Fig. S2**). The insertion of ST was confirmed by PCR using the overlap primers (**Fig. S2**). To test if EsxA-ST is expressed, the total lysate of Mm(EsxA-ST) was incubated with the purified SC-GFP and then applied to SDS-PAGE, followed by Western blot using either anti-EsxA antibody (Figure 4a) or anti-GFP antibody (Figure 4b). The results showed that EsxA-ST was successfully expressed, and a portion of EsxA-ST reacted with SC-GFP to form a higher molecular weight complex EsxA-ST-SC-GFP (~70 kDa) (Figure 4a **and B**). As a control, SC-GFP did not react with purified EsxA. Next, we tested if SC-GFP specifically reacts with the bacterial surface-associated EsxA-ST. The live mCherry-expressing Mm(EsxA-ST) and Mm(WT) were first incubated with SC-GFP, and then the cells were washed to remove residual SC-GFP, which was followed by fluorescence microscopy. We found that Mm(EsxA-ST) was labeled by SC-GFP and yield a significantly higher fluorescence overlap rate, but Mm(WT) was not, indicating that SC-GFP specifically recognized EsxA-ST and formed the EsxA-ST-SC-GPF complex on the cell surface (Figure 4c **and D**). Moreover, unlike EsxB-DAS4+, the expression and secretion of EsxA-ST were even higher than EsxA (Figure 4e). Not surprisingly, Mm(EsxA-ST) exhibit a strong intracellular survival in A549 cells, which was even stronger than Mm(WT) at 48 hpi (figure 4f), suggesting that ST doesn’t affect expression, secretion, and function of EsxA.

### SC-GFP inhibits the EsxA-ST-mediated liposome leakage and hemolysis of sheep red blood cells

**Figure 5.**
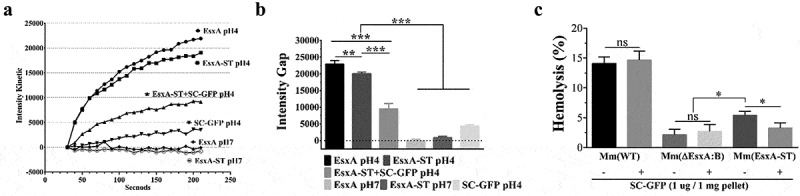
SC-GFP inhibited both the MPA of EsxA-ST and the bacteria-induced hemolysis of sheep RBCs. (a) The liposomes containing ANTS (fluorophore) and DPX (quencher) were incubated with the indicated proteins at either pH 7 or pH 4. The dequenching of ANTS fluorescence was recorded with times. (b) The relative fluorescence intensity change between 30 s and 210 s was calculated for each group. (c) Mm(WT), Mm(EsxA-ST) and Mm(ΔEsxA:B) were incubated with SC-GFP at RT, and then same bacterial amount of the strains with or without SC-GFP treatment were incubated with sheep RBCs to test hemolysis. The sheep RBCs incubated with PBS and Triton X-100 solution were used as the blank and positive control to calculate each strain’s hemolysis percentage. The experiments were replicated for three times, the data in curve graph is presented as mean, while the data in column graphs are presented as mean ± SD. For liposome leakage assay, statistical analysis was performed with One-way ANOVA method, followed by Holm-Sidak multiple comparison. For hemolysis assay, statistical analysis within each strain was performed with *t*-test. Statistical analysis between Mm(EsxA-ST) and Mm(ΔEsxA:B) was performed with One-way ANOVA method, followed by Holm-Sidak multiple comparison. *< 0.05, **P< 0.01, ***P< 0.001

Here, we determined the effect of SC-GFP on the MPA of EsxA-ST using the ANTS/DPX dequenching assay in liposome as previously described [19]. At pH 4, EsxA-ST induced less liposome leakage than EsxA, but it is still much higher than other groups, confirming that ST affects the MPA with a limited extent. However, in the presence of SC-GFP, the MPA of EsxA-ST was significantly inhibited, by 53% on average. As controls, both EsxA and EsxA-ST were not active in membrane permeabilization at pH 7 (Figure 5a **and B**). The result acquired with liposome only reflects SC-GFP’s effect on purified protein samples. To verify if SC-GFP could also inhibit the MPA of EsxA-ST at the contact interface of Mm(EsxA-ST) and mammalian cells, we tested the hemolytic ability of Mm(EsxA-ST) on sheep RBCs. In the hemolysis assay, upon incubation with SC-GFP, Mm(EsxA-ST) averagely decreased 39% hemolytic ability on sheep RBC, which further confirms that SC-GFP inhibits the MPA of EsxA-ST (Figure 5c). In contrast, the hemolytic activity of Mm(WT) was not affected by SC-GFP.

### Endogenous expression of SC-GFP reduces Mm(EsxA-ST) intracellular survival

**Figure 6.**
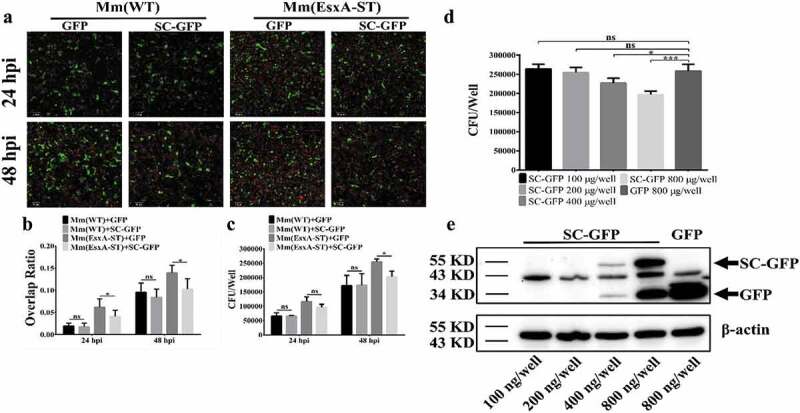
Endogenous expression of SC-GFP reduced Mm(EsxA-ST) intracellular survival in lung epithelial cells. (a) A549 cells were transiently transfected with pcDNA3.1-GFP or pcDNA3.1-SC-GFP. After 24 h of transfection, A549 cells were infected with Mm(WT) or Mm(EsxA-ST) at MOI = 2, respectively. At 24 and 48 hpi, the cells were fixed and images were taken at green channel and red channel to visualize GFP/SC-GFP and mCherry-expressing bacteria. For each strain, 12 random sights were taken from two replicate wells. Scale bar represents 50 μm. (b) The Red/Green overlap ratio of the cells in randomly selected fields was calculated to evaluate bacterial intracellular survival in transfect cells. (c) A549 cells were transiently transfected with pcDNA3.1-GFP or pcDNA3.1-SC-GFP. After 24 h of transfection, A549 cells were infected with Mm and Mm(EsxA-ST) at MOI = 2, respectively. At 24 and 48 hpi, the cells were harvested and subjected to CFU counting for intracellular survival. (d) A549 cells were transiently transfected with different concentrations of pcDNA3.1-SC-GFP DNA. After 24 h of transfection, A549 cells were infected with Mm(EsxA-ST) at MOI = 2. At 48 hpi, the cells were harvested and subjected to CFU counting for intracellular survival. The cells transfected with pcDNA3.1-GFP were used as the control. (e) The expression of SC-GFP in each transfection dose was detected with Western blots using anti-GFP antibody. The bands of SC-GFP and GPF are designated with arrows. β-actin was detected as a loading control. The intracellular survival experiment was replicated for three times and data is presented as mean ± SD. For the data in (**B**) and (**C**), statistical analysis was performed with multiple *t*-test between GFP and SC-GFP groups of each strain. *P< 0.05. For the data in (**D**), statistical analysis was performed with One-way ANOVA method, followed by Holm-Sidak multiple comparison. *P< 0.05, ***P< 0.001

To determine whether SC-GFP inhibits Mm(EsxA-ST) intracellular survival, we endogenously expressed SC-GFP by transient transfection in human type II lung epithelial cell A549 cells. Cellular fractionation analysis showed that the majority of SC-GFP is localized in the cytosol and a minor portion is localized in the membrane fraction (**Fig. S3**). The A549 cells were transiently transfected with SC-GFP for 24 h and then infected with Mm(EsxA-ST) and Mm(WT), respectively. Since both transfection efficiency and infection efficiency are less than 100% and varies, in order to obtain accurate and reliable results, we measured the intracellular survival with two independent approaches, fluorescence microscopy, and CFU counting. In the fluorescence microscopy method, we selected the cells containing both mCherry fluorescence (mycobacterial cells) and green fluorescence (either GFP or SC-GFP) and then quantified the Red/Green ratio in each cell (Figure 6a). The Red/Green ratio of the cells containing Mm(EsxA-ST) and SC-GFP was significantly lower than that of the cells containing Mm(EsxA-ST) and GFP (Figure 6b), while the Red/Green ratio of the cells containing Mm(WT) and SC-GFP was similar to the cells containing Mm(WT) and GFP (Figure 6b), suggesting that SC-GFP specifically inhibits Mm(EsxA-ST) intracellular survival. Consistent results were obtained in the CFU counting assay (Figure 6c), showing that at 48 hpi, the intracellular survival of Mm(EsxA-ST) in the cells expressing SC-GFP was significantly lower than that in the cells expressing GFP. To further verify that the decrease of Mm(EsxA-ST) intracellular survival is attributed to SC-GFP, we transfect A549 with different amounts of pcDNA3.1-SC-GFP. The result shows that as the expression of SC-GFP increased, Mm(EsxA-ST) exhibit a dose-dependent decrease of intracellular survival at 48 hpi, while the highest amount of pcDNA3.1-GFP has no effect on Mm(EsxA-ST) (Figure 6 **D and E**). This indicates that SC-GFP specifically inhibits Mm(EsxA-ST)’s intracellular survival in A549.

## Discussion

As a fish pathogen, Mm infection leads to tuberculosis-like diseases in fish and it often serves as a surrogate model organism for Mtb [32,54–56]. Genomic sequencing shows that although Mm’s genome is much larger than that of Mtb, many virulence genes, like *esxB/A*, share a high similarity in sequence and function between these two species [19,57,58]. It is well established that *esxB/A* contributes to Mm’s survival in mammalian cell lines [17,42,59]. In this study, two human lung epithelial cell lines WI-26 and A549 were used. It has been reported that lung epithelial cells are susceptible for Mtb infection and contributes to Mtb colonization [60–62]. Deletion of EsxA/B results in attenuated infection in lung epithelial cells [63], suggesting that the lung epithelial cells were suitable for functional studies of EsxA/B in Mm infection. However, further studies should be performed with *M. tuberculosis* to confirm the obtained results.

Despite of extensive studies in the past decades, the data regarding the role of EsxA in mycobacterial pathogenesis, particularly in phagosome rupture and cytosolic translocation, is still conflicting. The artifacts generated from gene knockout may be a major reason for the discrepancy and confusion. Deletion of EsxAB will affect other factors (e.g. EspA, EspB, and EspC) that are codependently secreted with EsxAB [18,23–26]. And it has been reported that deletion of specific genes or locus leads to global disturbance of gene expression [27–29]. Therefore, to avoid the potential artifacts induced by gene deletion, we engineered the Mm(EsxB-DAS4+) strain for inducible knockdown of EsxB at the post-translational level (**Fig. S1**). The DAS4+ system is based on mycobacterial innate protease ClpXP to degrade the intracellular protein [30,31]. DAS4+ tag is only found in *E.coli*, and its degradation efficiency depends on SspB, a ClpXP adaptor that has no homologies in mycobacteria. In this way, the inducible SspB expression makes the DAS4+-tagged mycobacterial protein be degraded by ATC induction [31,64]. Initially, the DAS4+ tag was attached to C-terminus of EsxA, but no positive colonies were acquired, so we engineered Mm(EsxB-DAS4+). Compared to Mm(WT), Mm(EsxB-DAS4+) had a lower expression and secretion, hence a lower intracellular survival (Figure 1a). When comparison was performed between Mm(ΔEsxA:B) and Mm(EsxB-DAS4+), Mm(EsxB-DAS4+) still exhibited a significantly higher intracellular survival, which is probably due to the presence of EsxA and EsxB-DAS4+ (Figure 1b). Unsurprisingly, ATC induction also caused degradation of EsxA, which is consistent with the fact that EsxB and EsxA form a heterodimer [49,50]. Moreover, ATC didn’t affect the mRNA level of EsxB, indicating that the ATC-induced knockdown of EsxB (also EsxA) occurs at the post-translational stage.

As expected, inducible knockdown of EsxB significantly attenuated Mm intracellular survival, to a level that is similar to Mm(ΔEsxA:B) (Figure 3a). In Figure 3c **and D**, ATC was added after mycobacterial entry into the cells to knockdown EsxB-DAS4+ expression during the process of infection. It has been reported that in THP-I cells, Mm requires the ESX-1 system to escape from the phagosome for intracellular replication, which occurs at around 20 hpi [42]. In consistent with the reported timeline of infection, our result showed that knockdown of EsxB-DAS4+ led to a decrease in intracellular survival at 24 hpi. Mm(WT) exhibited a decreased intracellular survival, but the decrease is much smaller than that of the ATC-treated Mm(EsxB-DAS4+). Thus, the decrease in intracellular survival of Mm(WT) could be due to nonspecific antibiotic effect of ATC.

However, there is still a concern that inducible knockdown of EsxB-DAS4+ (also EsxA) affects secretion of other codependent factors. Thus, we switched the strategy from expression to functional inhibition of the MPA of EsxA at the post-secretional level by using the Spy-Tag/Spy-Catcher system [51]. The system is based on spontaneous formation of an amide linkage between Lys and Asn, resulting in an intramolecular isopeptide bond in the pilin protein in *Streptococcus pyogenes* [65,66]. After optimization, Spy-Tag was engineered to be a 13aa peptide that binds fast and stably with the 138aa Spy-Catcher under various conditions [52,53]. Unlike antibody-antigen interaction, the covalent linkage between ST and SC promotes its usage for protein-protein interaction, protein modification, and fluorescent imaging [67,68].

In this study, EsxA-ST was properly expressed and secreted. Since the live Mm(EsxA-ST) could be labeled with SC-GFP (Figure 4), it supports that EsxA resides on bacterial surface [62,69,70]. Obviously, SC-GFP is only able to bind and react with EsxA-ST after secretion and has no effect on EsxB’s secretion (**Fig. S5**). According to current ESX-1 system model [71], once EsxA-ST is secreted out of mycobacterial membrane, SC-GFP is unlikely to affect ESX-1 substrates, which minimizes the potential artifacts brought by genetic deletion. However, since the interaction between ESX-1 substrates is not fully studied, further experiment like mass spectrometry-based proteomic would provide more details about SC-GFP’s effect on ESX-1 [25]. In liposome leakage assay, EsxA-ST’s MPA is lower than that of EsxA, while Mm(EsxA-ST)’s survival in A549 was not affected. This indicates ST attachment does not impact EsxA-ST’s normal function. However, it naturally intrigued us to wonder if a larger attachment like SC-GFP would result in great impact to serve our strategy. As we expected, SC-GFP significantly inhibits the MPA of EsxA-ST in the liposome leakage assay (Figure 5 **A and B**) as well as the Mm(EsxA-ST)-induced hemolysis of sheep RBCs (Figure 5c). We notice that while Mm(EsxA-ST) had a higher intracellular survival in A549 than Mm(WT) (figure 4f), but Mm(EsxA-ST) induced a lower hemolysis of sheep RBCs than Mm(WT) (Figure 5c). This is largely because Mm(EsxA-ST) forms more clumps in 7H9 media. Even after being pushed through a 27-Gauge 1/2 inch needle for several times, there were still many clumps. In comparison, Mm(WT) was mixed with RBCs homogeneously, while some Mm(EsxA-ST) cell clumps were aggregated at the bottom of tubes, which decreased the overall Mm(EsxA-ST)’s hemolytic efficiency. Nevertheless, the exogenously added SC-GFP was still able to inhibit the hemolysis of Mm(EsxA-ST), strongly indicating that SC-GFP reacts with EsxA-ST and inhibits the MPA of EsxA-ST.

Endogenous expression of SC-GFP in A549 cells significantly downregulated Mm(EsxA-ST) intracellular survival (Figure 6). Since the rate of transient transfection and the rate of infection never reached 100% in the target cells, there were cells that were transfected but not infected and vice versa. In order to accurately measure the effect of SC-GFP expression on mycobacterial infection, we applied two independent approaches, fluorescence quantification, and CFU, as described in Figure 6. The fluorescence quantification was to calculate red/green ratio in the cells with both red (mCherry) and green fluorescence (SC-GFP or GFP). However, the CFU approach was not able to distinguish the heterogeneity of the cells. Because the cells that were infected but not transfected by SC-GFP were included in the CFU counting, the SC-GFP specific inhibitory effect was diluted, which may explain why at 24 hpi SC-GFP didn’t show significant downregulation on Mm(EsxA-ST) CFU (Figure 6c). To further test the SC-GFP inhibitory effect on Mm(EsxA-ST), A549 cells were transfected with different amounts of pcDNA3.1-SC-GFP. The results showed that Mm(EsxA-ST) intracellular survival was negatively correlated with SC-GFP expression (Figure 6d **and E**). Combined with the results in Figure 5, it strongly indicates that SC-GFP inhibits Mm(EsxA-ST) intracellular survival by inhibiting the MPA of EsxA-ST.

Although the overall inhibitory effect of SC-GFP on Mm(EsxA-ST) intracellular survival was significant when compared with GFP, the rate of inhibition was only ~30% (Figure 5). The limited inhibitory effect of SC-GFP could be attributed to the following reasons. First, EsxA has been implicated to crosstalk with other cellular organelles and immune signal pathways [17,72,73], thus SC-GFP might not affect its function other than MPA. Second, it is probably that EsxA is not sufficient for mycobacterial phagosome rupture and cytosolic translocation solely, other factors may also contribute to this process. It has been reported that EsxA works with phthiocerol dimycocerosates (PDIM) to mediate phagosome rupture [74,75], and PDIM alone could maintain certain level of hemolysis with Mm [76]. Also, deletion of other ESX-1 substrates would impact mycobacteria phagosome escape ability [17,38]. A recent study has reported that deletion of *espE/F* significantly inhibits Mm’s hemolysis, while the expression and secretion of EsxA/B were not affected [77], indicating that EsxA and EsxB are not sufficient for mycobacteria lysis-related phenotype. We also notice that deletion of *espE* or *espF* upregulates the expression and secretion of EsxA and EsxB, which provides an evidence that genetic deletion may introduce artifacts by altering expression of other genes. Thus, the DAS4+ system and ST-SC system utilized in this manuscript have advantages and novelty in minimizing potential artifacts as compared to genetic knockout. In future, more sensitive approaches, like mass spectrometry, may need to be applied to investigate the other factors that may be involved in the process [25]. Third, the inhibition efficiency is also affected by subcellular localization of SC-GFP. The endogenously expressed SC-GFP is mainly localized in the cytosol with only a small part of SC-GFP localized in the membrane fraction (**Fig. S3**). Current studies suggest a model that mycobacteria are internalized into the phagosomes and EsxA mediates mycobacteria to escape from the phagosome and translocate to the cytosol for replicating and cell-to-cell spreading [42,59,75]. Therefore, we propose that SC-GFP may have limited access to the EsxA-ST within the phagosome, if SC expression could be directed into phagosome, the downregulation of Mm(EsxA-ST) intracellular survival would be more significant. Moreover, according to previous reports, lack or downregulation of EsxA would lead to attenuated mycobacterial intracellular survival, as well as virulence in susceptible hosts [21,63,78]. If we use genetically modified animal models to test SC’s effect on Mm(EsxA-ST), it would be more convincing that EsxA contributes to mycobacterial virulence with its MPA.

In summary, by using DAS4+ system and ST/SC system we were able to knockdown EsxB (also EsxA) and inhibit EsxA MPA at the post-translational level. The results unambiguously support the direct role of EsxA MPA in mycobacterial intracellular survival. The two systems can be powerful tools in studies of host-pathogen interaction, gene function, protein-protein interaction, and protein intracellular trafficking, etc.

## Supplementary Material

Supplemental Material
