# Supplementary material for: Post-translational knockdown and post-secretional modification of EsxA determine contribution of EsxA membrane permeabilizing activity for mycobacterial intracellular survival
Source: Virulence. 2021 Jan 11;12(1):312–28. doi: 10.1080/21505594.2020.1867438 (PMC7808419; doi:10.1080/21505594.2020.1867438)
Supplement: Supplemental Material [file KVIR_A_1867438_SM3840.docx]

**Fig.S1. Construction and identification of the Mm(EsxB-DAS4+) strain.** (**A**) The suicide plasmid pJSC407-sacB was used to insert DAS4+ to the C-terminus of EsxB. Then the pGMCKq1-10M1-ssBopt plasmid that encodes adaptor protein was electroporated into Mm(EsxB-DAS4+). (**B**) The heated culture pellet of Mm(EsxB-DAS4+) was applied for PCR to confirm the insertion of DAS4+. The primers that overlap the insert sequence were used and produced a specific DNA fragment with an expected length of 378 bp, which is absent in the heated culture pellet of Mm(WT). The suicide plasmid pJSC407-sacB was used as positive control. 2-Log DNA Ladder (NEB, Ipswich, MA, USA) was used as molecular marker. The nucleic electrophoresis was conducted with 1% agarose gel, which contains 1μg/ml EtBr for imaging.

**Fig.S2. Construction and identification of the Mm(EsxA-ST) strain.** (**A**) The suicide plasmid pJSC407-sacB was used to insert ST to the C-terminus of EsxA. (**B**) The heated culture pellet of Mm(EsxA-ST) was applied for PCR to confirm the insertion of ST. The primers that overlap the insert sequence were used and produced a specific DNA fragment with an expected length of 263 bp, which is absent in the heated culture pellet of Mm(WT). The suicide plasmid pJSC407-sacB was used as positive control. 2-Log DNA Ladder (NEB, Ipswich, MA, USA) was used as molecular marker. The nucleic electrophoresis was conduct with 1% agarose gel, which contains 1μg/ml EtBr for imaging.

**Fig.S3. Subcellular localization of SC-GFP in A549 cells.** The A549 cells were transfected with pcDNA3-SC-EGFP or pcDNA3-EGFP for 24 h and then the cells were harvested, lysed and fractionated into post-nuclear supernatant (PNS), cytosolic fraction and membrane fraction. The samples were applied to SDS-PAGE, followed by Western blots using anti-GFP antibody.

**Fig.S4. The IFA detection of mycobacteria-associated EsxA on Mm(EsxB-DAS4+)|pGMCKq1.** (**A**)The mCherry-expressing Mm(EsxB-DAS4+)|pGMCKq1 cells were treated with or without ATC (0.5 μg/ml) for 48 h. Then the bacteria were incubated with anti-EsxA serum, followed by FITC-labeled secondary antibody, to detect the bacteria-associated EsxA. Images from all groups were taken under a LSM700 confocal fluorescence microscopy with the same configuration. For each strain, 12 random sights were taken from two replicate wells. The scale bar represents 50 µm. (**B**) The Green/Red overlap ratio in the randomly selected sights was quantified. The left fragment of X axis ranges from 0 to 0.008, and the right fragment ranges from 0.04 to 0.5. The IFA assay were replicated for three times and the data is presented as mean ± SD. The statistical analysis was performed with One-way ANOVA method, followed by Holm-Sidak multiple comparison. ^****^*P*<0.0001.

**Fig.S5. Western blot detection of EsxB secretion in SC-GFP-treated Mm(EsxA-ST) culture filtration.** SC-GFP was added into Sauton media of Mm(EsxA-ST) at 1 μg/ml and the culture filtration was collected 72 h later by centrifugation. EsxB was detected with anti-EsxB serum. EsxA and EsxA-SC-GFP were detected with anti-EsxA antibody. Ag85B was detected with anti-Ag85B serum as a control.


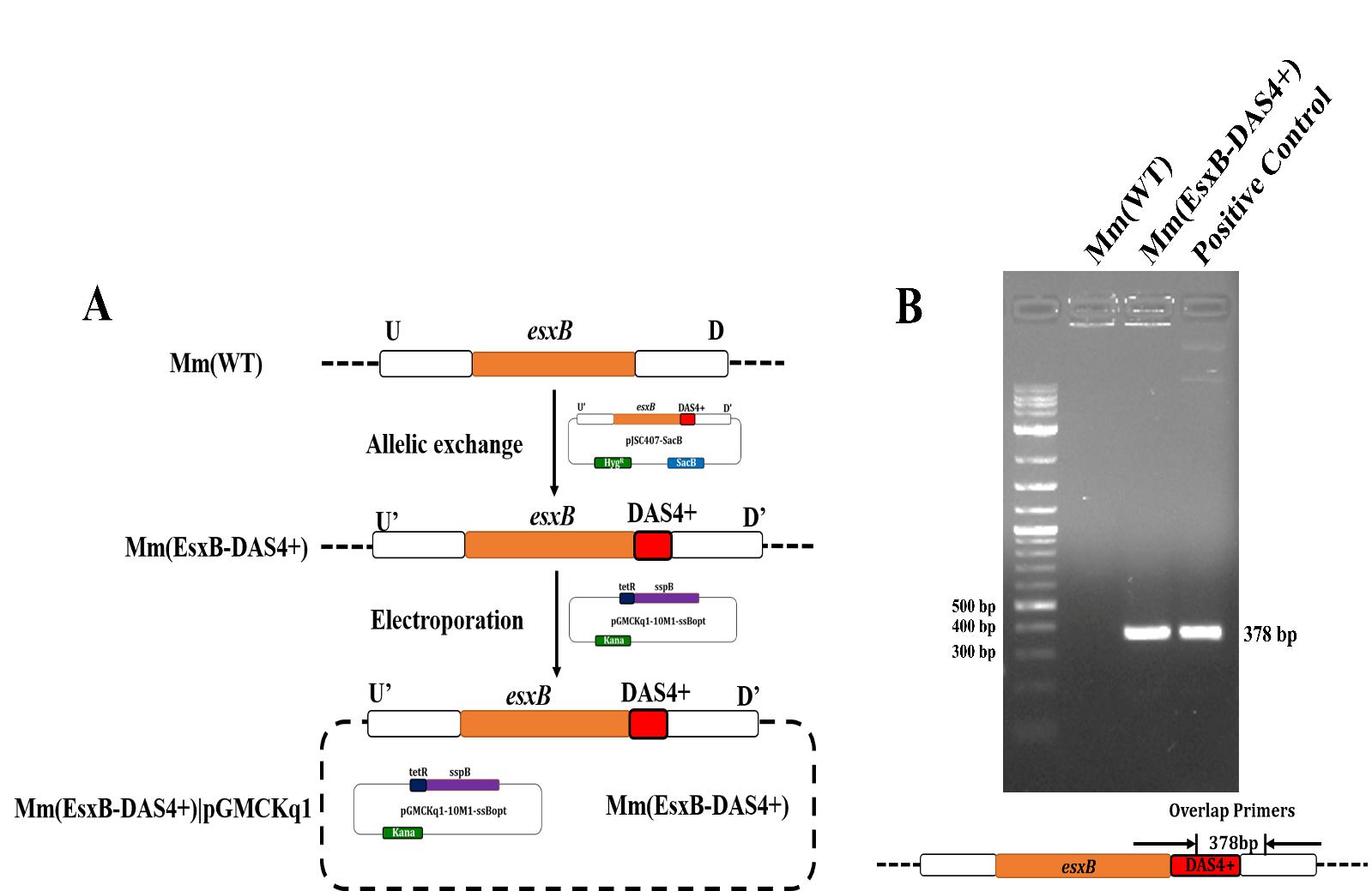


Figure S1


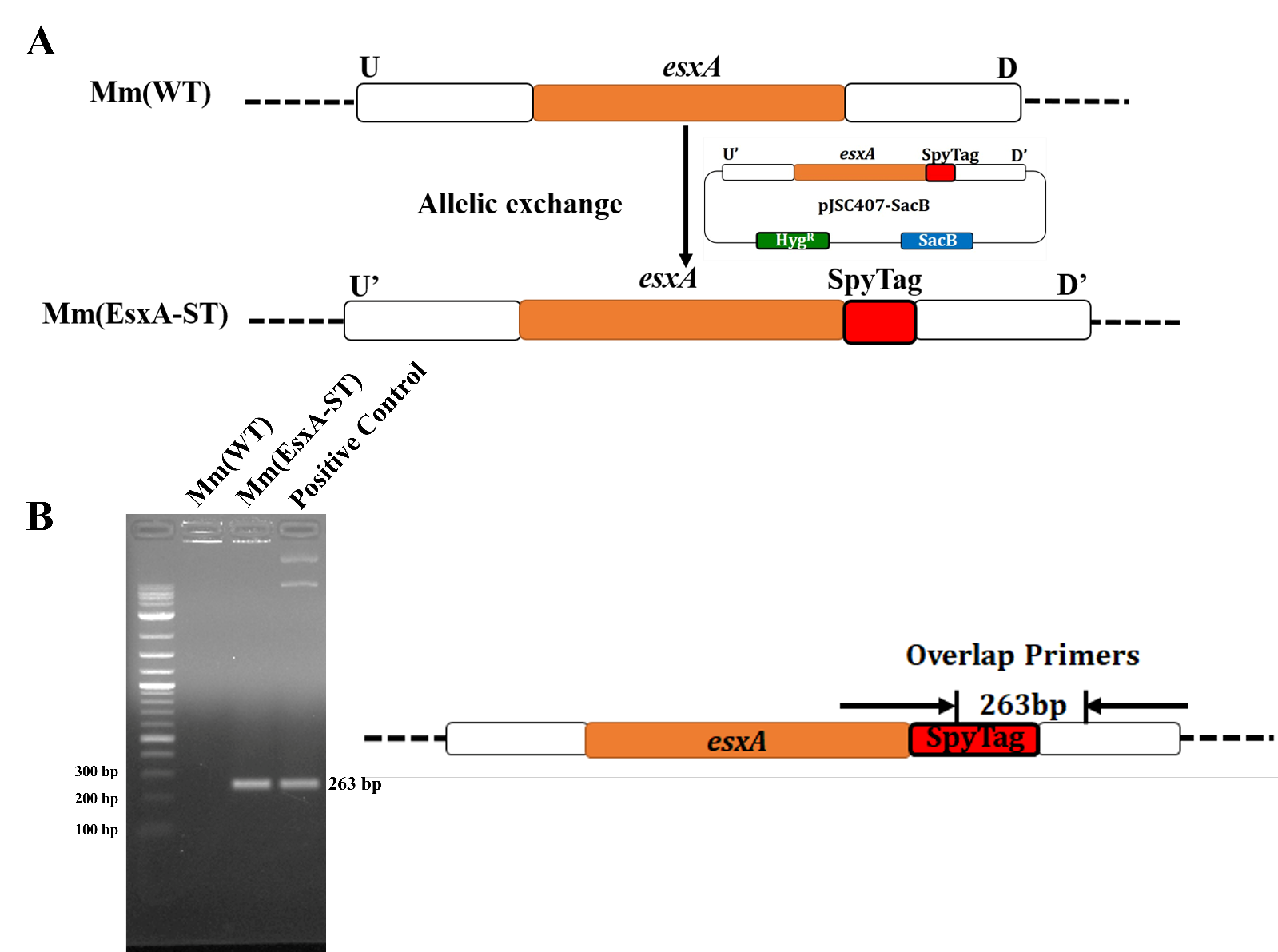


Figure S2


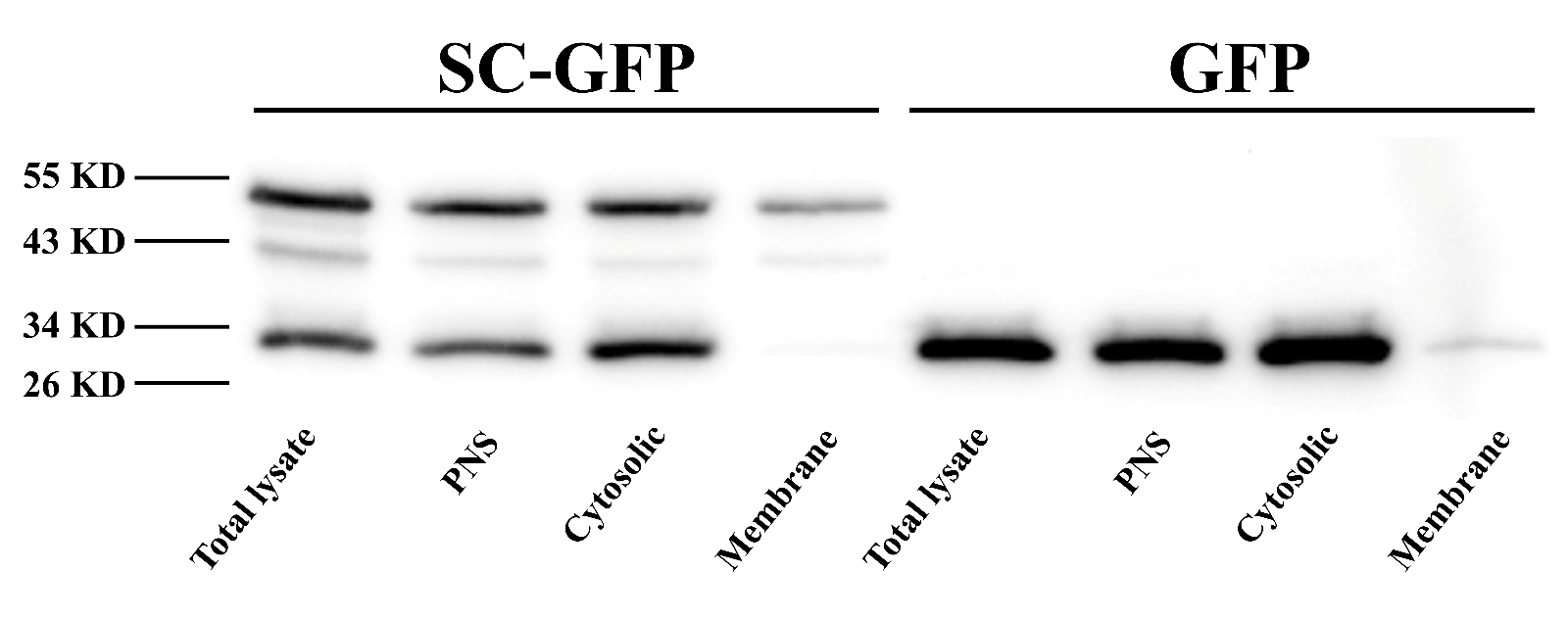


Figure S3


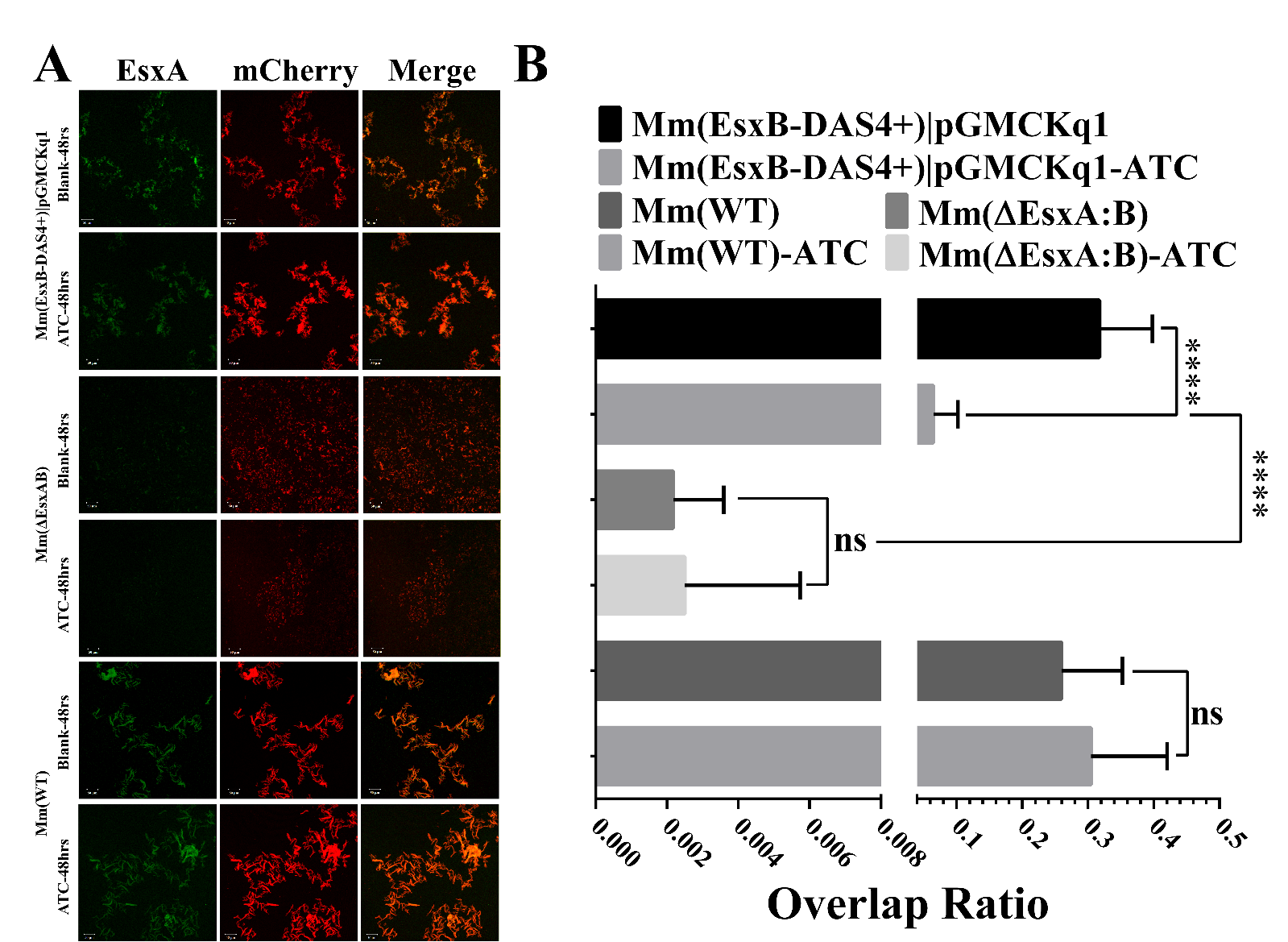


Figure S4


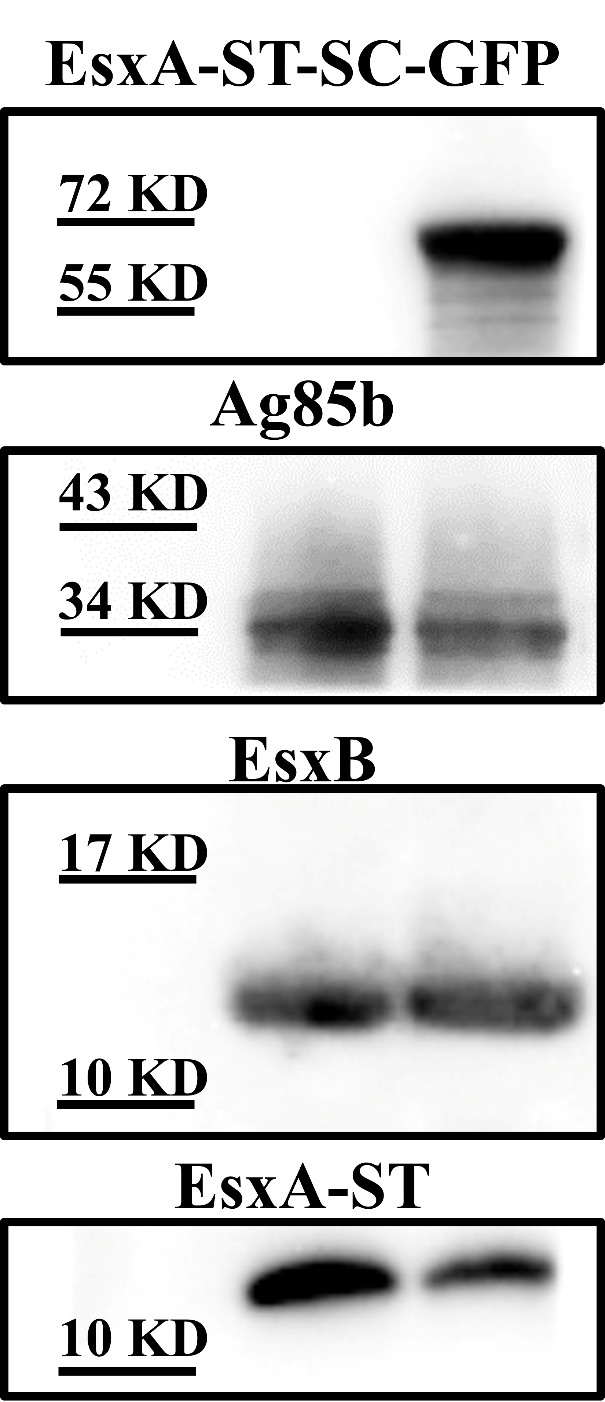


Figure S5
